# Metabolome‐Genome‐Wide Association Study (mGWAS) Reveals Novel Metabolites Associated with Future Type 2 Diabetes Risk and Susceptibility Loci in a Case‐Control Study in a Chinese Prospective Cohort

**DOI:** 10.1002/gch2.202000088

**Published:** 2021-03-23

**Authors:** Yang Ouyang, Gaokun Qiu, Xinjie zhao, Benzhe Su, Disheng Feng, Wangjie Lv, Qiuhui Xuan, Lichao Wang, Di Yu, Qingqing Wang, Xiaohui Lin, Tangchun Wu, Guowang Xu

**Affiliations:** ^1^ CAS Key Laboratory of Separation Science for Analytical Chemistry Dalian Institute of Chemical Physics Chinese Academy of Sciences 457 Zhongshan Road Dalian 116023 China; ^2^ University of Chinese Academy of Sciences Beijing 100049 China; ^3^ MOE Key Lab of Environment and Health School of Public Health Tongji Medical College Huazhong University of Science & Technology Wuhan Hubei 430030 China; ^4^ School of Computer Science & Technology Dalian University of Technology Dalian 116024 China

**Keywords:** genomics, mGWAS, nested case‐control study, type 2 diabetes, untargeted metabolomics

## Abstract

In a Chinese prospective cohort, 500 patients with new‐onset type 2 diabetes (T2D) within 4.61 years and 500 matched healthy participants are selected as case and control groups, and randomized into discovery and validation sets to discover the metabolite changes before T2D onset and the related diabetogenic loci. A serum metabolomics analysis reveals that 81 metabolites changed significantly before T2D onset. Based on binary logistic regression, eight metabolites are defined as a biomarker panel for T2D prediction. Pipecolinic acid, carnitine C14:0, epinephrine and phosphatidylethanolamine 34:2 are first found associated with future T2D. The addition of the biomarker panel to the clinical markers (BMI, triglycerides, and fasting glucose) significantly improves the predictive ability in the discovery and validation sets, respectively. By associating metabolomics with genomics, a significant correlation (*p* < 5.0 × 10^−8^) between eicosatetraenoic acid and the FADS1 (rs174559) gene is observed, and suggestive correlations (*p* < 5.0 × 10^−6^) between pipecolinic acid and CHRM3 (rs535514), and leucine/isoleucine and WWOX (rs72487966) are discovered. Elevated leucine/isoleucine levels increased the risk of T2D. In conclusion, multiple metabolic dysregulations are observed to occur before T2D onset, and the new biomarker panel can help to predict T2D risk.

## Graphical abstract

In a Chinese prospective cohort, 500 patients with new‐onset type 2 diabetes (T2D) within 4.61 years and 500 matched healthy participants are selected. A serum metabolomics analysis is performed and 81 metabolites are found to significantly change before T2D onset. The new biomarker panel helps to predict T2D. Gene FADS1, CHRM3, and WWOX are found to be associated with T2D‐related metabolites.

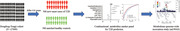

## 1 Introduction

Type 2 diabetes (T2D) is a prevalent, progressive chronic disease that increases the risk of heart disease and microvascular complications.^[^ ^1^ ^]^ The occurrence of T2D can be prevented by controlling diet and increasing exercise. Thus, early diagnosis and early intervention are very meaningful for patients. Although some clinical biomarkers are characteristic of diabetes (e.g., glucose and HbA1c), biomarkers for predicting diabetes are still urgently needed.

Metabolomics, which focuses on endogenous small‐molecule metabolites, is becoming a reliable tool for discovering potential biomarkers and explaining the pathogenesis of diseases.^[^ ^2^ ^]^ In prospective nested case‐control studies, cases and controls are from the same cohort, and selection bias in effect estimation can be reduced. Recently, prospective nested case‐control studies have been used to reveal the changes in the metabolome antecedent to diabetes and to find predictors of future diabetes.^[^ ^3^, ^4^, ^5^ ^]^ In a European nested case–control study, several glycerophospholipids, sugar metabolites and a purine nucleotide were found to be significantly changed before the onset of T2D.^[^ ^3^ ^]^ In addition, alanine, phenylalanine, tyrosine, and palmitoylcarnitine were also found to be risk factors for incident T2D in two independent Chinese prospective cohort studies based on a targeted metabolomics method.^[^ ^4^ ^]^ In another Chinese cohort study, 38 lipids were found to be significantly associated with the risk of T2D. Then, the combination of six lipids and eight clinical parameters improved the predictive ability of the clinical parameters from 0.710 to 0.781 in the discovery set and from 0.698 to 0.722 in the validation set.^[^ ^5^ ^]^

T2D is a metabolic disease affected by multiple genes and has a strong genetic predisposition. Therefore, the discovery of genetic loci associated with T2D may help understand the pathogenesis. Recently, a metabolome‐genome‐wide association study (mGWAS) was used to verify known gene loci and identify new related gene loci. In a nested case‐control study of a Korean population, nine metabolites were found to be significantly related to the occurrence of diabetes.^[^ ^6^ ^]^ These T2D‐related metabolites were used as intermediate molecular phenotypes in mGWAS to link genes and incident T2D. CPS1 (rs1047883), which is significantly associated with glycine, was shown to be related to the risk of T2D. The newly discovered genetic loci and their relationships with metabolites can help understand the complex pathogenesis of T2D. However, to the best of our knowledge, no mGWAS of T2D has been conducted in Chinese prospective nested case‐control cohorts so far.

In this work, a prospective nested case‐control study was conducted in the Dongfeng‐Tongji cohort^[^ ^7^ ^]^ of the Chinese population. The case group of this study consisted of 500 participants who developed T2D after an average follow‐up of 4.61 ± 0.15 years, and each case was matched with a nondiabetic participant in the cohort to form the control group. Serum untargeted metabolomics analysis was performed to identify significantly differential metabolites before the onset of T2D, and a new combinational metabolite marker panel was established to predict T2D in advance. Then, metabolomics and genomics were integrated to reveal possible diabetogenic loci and causal relationships between metabolites and T2D.

## 2 Results

### 2.1 Baseline Characteristics

Baseline characteristics were compared between the case group and control group in both the discovery and validation sets (**Table** **1**). As shown in Table 1, there was no significant difference in age, sex, smoking status, drinking status, or physical activity in the discovery set between the case and control groups. The case group had a significantly higher body mass index (BMI), systolic blood pressure (SBP), diastolic blood pressure (DBP), triglyceride (TG) level, and fasting glucose (FG) level and a lower high‐density lipoprotein (HDL) cholesterol level than the control group (*p* < 0.05, false positive rate (FDR) < 0.1). The odds ratios (ORs) per standard deviation (SD) increment in clinical parameters were calculated by Cox regression (Table S1, Supporting Information). For the significantly different clinical parameters, the risk of T2D was significantly positively correlated with baseline BMI, SBP, DBP, TG, and FG levels (the ORs per SD increment were greater than 1) and was significantly negatively correlated with HDL level (the OR per SD increment was less than 1). For the validation cohort, the BMI, TG, and FG levels were significantly higher in the participants in the case group than in those in the control group, and these parameters were significantly positively correlated with the risk of T2D.

**Table 1:** Baseline characteristics of participants in the discovery set and validation set

Variables	Discovery set				Validation set			
	Controls (*n* = 292)	Cases (*n* = 292)	*p* ^a)^	FDR^a)^	Controls (*n* = 208)	Cases (*n* = 208)	*p* ^a)^	FDR^a)^
Age (years)	62.38 ± 7.05	62.34 ± 7.10	0.993	1.000	62.52 ± 7.44	62.47 ± 7.46	0.946	1.000
Men sex, No. (%)	47.3%	47.3%	1.000	1.000	40.9%	40.9%	1.000	1.000
BMI (kg m^−2^)	24.16 ± 3.31	25.96 ± 3.65	<0.001	<0.001	23.68 ± 3.02	25.64 ± 3.09	<0.001	<0.001
Smoking status, No. (%)								
Current smoker	24.8%	20.6%	0.400		23.2%	18.9%	0.450	
Former smoker	9.7%	11.3%		0.486	6.3%	7.8%		0.679
Never smoker	65.5%	68.0%			70.5%	73.3%		
Drinking status, No. (%)								
Current drinker	27.1%	26.7%	0.768		22.6%	19.2%	0.322	
Former drinker	3.4%	5.5%		0.870	5.8%	4.8%		0.548
Never drinker	69.5%	67.8%			71.6%	76.0%		
Physical activity, No. (%)	90.8%	87.3%	0.186	0.263	88.5%	87.0%	0.654	0.833
Systolic blood pressure (mmHg)	127.00 ± 18.67	130.64 ± 17.93	0.015	0.028	127.07 ± 17.27	129.54 ± 18.54	0.196	0.417
Diastolic blood pressure (mmHg)	76.48 ± 11.07	79.04 ± 11.08	0.010	0.021	77.30 ± 10.19	77.91 ± 10.56	0.480	0.679
HDL cholesterol (mmol L^−1^)	1.49 ± 0.45	1.40 ± 0.49	0.002	0.004	1.46 ± 0.43	1.38 ± 0.39	0.037	0.089
LDL cholesterol (mmol L^−1^)	3.01 ± 0.79	3.05 ± 0.72	0.333	0.436	3.01 ± 0.78	2.99 ± 0.74	0.909	1.000
Triglycerides (mmol L^−1^)	1.29 ± 0.70	1.64 ± 0.93	<0.001	<0.001	1.36 ± 0.83	1.67 ± 1.00	<0.001	<0.001
Fasting glucose (mmol L^−1^)	5.53 ± 0.55	5.99 ± 0.61	<0.001	<0.001	5.51 ± 0.58	6.02 ± 0.56	<0.001	<0.001

^a)^ Values of *p* and FDR were calculated by nonparametric tests.

### 2.2 Significantly Changed Metabolites and Pathways Related to T2D Onset

**Figure 1:**
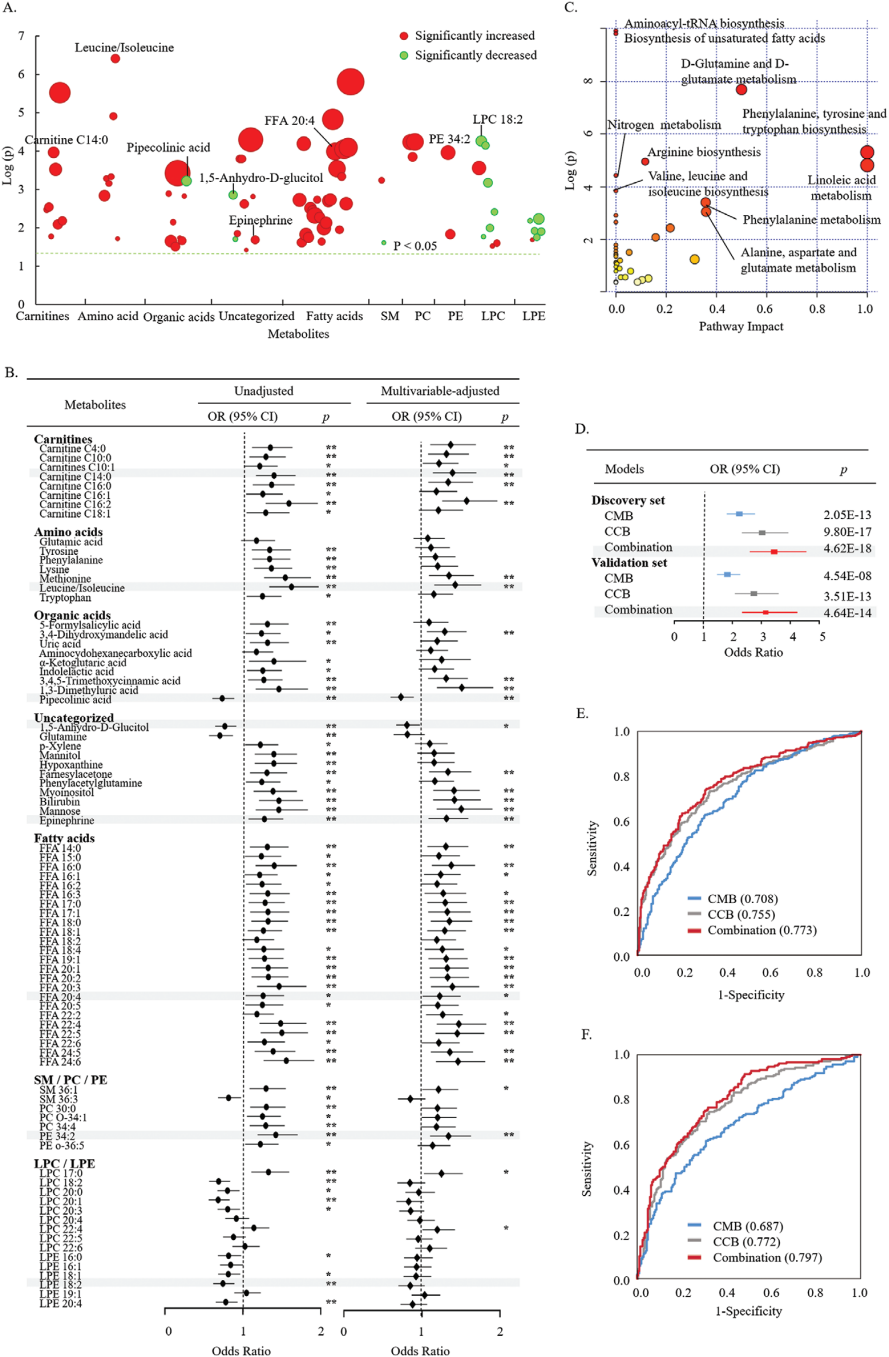
A) Scatter plot of significantly changed metabolites whose *p* values were below 0.05 and FDR values were below 0.1 in the discovery set. The diameter of the circles indicates the degree of metabolite changes. Significantly changed metabolites with an increased level in the case group are marked in red. Significantly changed metabolites with a decreased level in the case group are marked in green. B) Significantly changed metabolites and the risk of diabetes in the discovery set. Unadjusted ORs per SD increment and multivariable‐adjusted ORs per SD increment are shown (*: *p* < 0.05; **: *p* < 0.01). The multivariable‐adjusted ORs were adjusted by age, sex, BMI, smoking status, drinking status, and physical activity. C) Pathway analysis based on significantly changed metabolites before diabetes onset. D) ORs per SD increment in predictive model scores of T2D. ROC curves of the discovery set E) and validation set F). CMB consisted of eight metabolites, and CCB consisted of BMI, TG and FG. The combination was composed of CMB and CCB.

Serum samples of the discovery set were analyzed by a liquid chromatography‐mass spectrometry (LC‐MS)‐based metabolomics approach. A total of 206 metabolites were identified according to our in‐house database.^[^ ^8^ ^]^ Among these identified metabolites, 94.7% of the metabolites had a relative standard deviation (RSD) value less than 30% among quality control (QC) samples in the discovery set (Figure S1A, Supporting Information) and were used for the following data analysis. Nonparametric tests were conducted to define significantly changed metabolites. **Figure** **1**A shows the changes of 81 significantly differential metabolites whose *p* values were below 0.05 and FDR values were below 0.1. To study the relationship between differential metabolites and future T2D risk, the ORs per SD increment were calculated (Figure 1B). The multivariable‐adjusted ORs were adjusted by matched factors (age, sex), obesity (BMI) and lifestyle factors (smoking status, drinking status and physical activity). As shown in Figure 1A,B, the levels of acylcarnitines, branched‐chain amino acids, aromatic amino acids, free fatty acids (FFAs), phosphatidylcholines (PCs) and phosphatidylethanolamines (PEs) significantly increased before T2D occurred, and those metabolites were significantly positively correlated with the risk of future T2D (values of ORs greater than 1). Most acylcarnitines and fatty acids remained significant after multivariable adjustment. On the other hand, the levels of most lysophosphatidylethanolamines (LPEs) and lysophosphatidylcholines (LPCs) decreased, and they were significantly negatively correlated with the risk of future T2D (values of ORs less than 1). In addition, the results of pathway analysis based on significantly changed metabolites are shown in Figure 1C. Aminoacyl‐tRNA biosynthesis (*p* < 0.001, FDR = 0.002) and biosynthesis of unsaturated fatty acids (*p* < 0.001, FDR = 0.002) were the most significantly altered pathways before T2D onset.

### 2.3 Future Risk‐Related Metabolites and Metabolic Markers for T2D Prediction

To distinguish the control group from the case group, we performed binary logistic regression on the 81 metabolites that were significantly different between the case group and control group in the discovery set. A combinational metabolic biomarker panel containing eight metabolites was established, including pipecolinic acid, 1,5‐anhydro‐D‐glucitol, LPC 18:2, carnitine C14:0, leucine/isoleucine, eicosatetraenoic acid (FFA 20:4), epinephrine and PE 34:2. For convenience, they are named combination metabolite biomarker (CMB). The change trends of these eight metabolites are shown in Figure 1A, and the distribution of ORs is shown in Figure 1B and Table S2 (Supporting Information). Compared with those in the control group, the levels of carnitine C14:0, leucine/isoleucine, FFA 20:4, epinephrine and PE 34:2 increased in the case group, and their baseline levels were significantly positively correlated with the risk of T2D. The multivariable‐adjusted ORs per SD increment were 0.731 (95% CI 0.597–0.896, *p* = 0.002) for carnitine C14:0, 1.427 (1.157–1.761, *p* < 0.001) for leucine/isoleucine, 1.229 (1.009–1.497, *p* = 0.041) for FFA 20:4, 1.315 (1.084–1.595, *p* = 0.005) for epinephrine, and 1.341 (1.103–1.631, *p* = 0.003) for PE 34:2. Furthermore, before the onset of T2D, the levels of pipecolinic acid, 1,5‐anhydro‐D‐glucitol and LPC 18:2 decreased, and their baseline levels were negatively associated with future T2D risk. The multivariable‐adjusted ORs per SD increment were 0.731 (0.597–0.896, *p* = 0.002) for pipecolinic acid and 0.808 (0.670–0.973, *p* = 0.025) for 1,5‐anhydro‐D‐glucitol. For LPC 18:2, the unadjusted OR per SD increment was 0.690 (0.569–0.837, *p* < 0.001), but it became insignificant after multivariable adjustment (*p* = 0.116).

In addition, binary logistic regression was also performed on significantly different clinical parameters in the discovery set. BMI, TG, and FG were defined as a combinational clinical biomarker panel (CCB). The ORs per SD increase in T2D were 3.028 (95% CI 2.332–3.933, *p* < 0.001) for CCB, 2.243 (95% CI 1.808–2.782, *p* < 0.001) for CMB and 3.445 (95% CI 2.604–4.558, *p* < 0.001) for the combination model (CMB + CCB) (Figure 1D). The areas under the receiver operating characteristic curves (AUCs) of CMB and CCB were 0.708 and 0.755, respectively. Adding CMB to CCB significantly improved the predictive ability (AUC = 0.773, *p* = 0.031) (Figure 1E).

To validate these three predictive models, 208 pairs of cases and matched control samples were used as the validation set. Serum samples in the validation set were analyzed by the same LC‐MS‐based metabolomics approach, and 89.8% of the 206 identified metabolites had an RSD value of less than 30% in the QC samples (Figure S1B, Supporting Information). The ORs per SD increase in CMB, CCB and their combination were 1.832 (95% CI 1.475–2.276), 2.742 (95% CI 2.090–3.599) and 3.150 (95% CI 2.338–4.244), respectively, and all *p* values of the ORs were less than 0.001 (Figure 1D). The AUCs of CMB and CCB were 0.687 and 0.772, respectively (Figure 1F). Adding CMB to CCB significantly improved the predictive ability (AUC = 0.797, *p* = 0.017).

### 2.4 Susceptibility Loci and Causal Relationships

**Figure 2:**
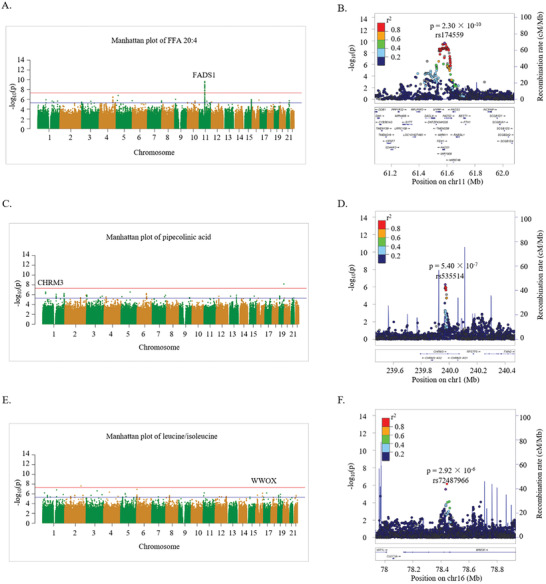
A) Manhattan plot of FFA 20:4. B) Regional plot showing LD (*r* ^2^) and *p* values of FFA 20:4‐related SNPs near the FADS1 gene. C) Manhattan plot of pipecolinic acid, D) Regional plot showing LD (*r* ^2^) and *p* values of pipecolinic acid‐related SNPs near the CHRM3 gene. E) Manhattan plot of leucine/isoleucine. F) Regional plot showing LD (*r* ^2^) and *p* values of leucine/isoleucine‐related SNPs near WWOX gene. The blue line and red line indicate the suggestive (*p* < 5.0 × 10^−6^) and significant (*p* < 5.0 × 10^−8^) genome‐wide thresholds, respectively.

On the basis of the above study, we conducted mGWAS between significantly changed metabolites at baseline and SNPs to find the potential locus related to incident T2D. The GWAS results of FFA 20:4 are shown in **Figure** **2**. Figure 2A is the Manhattan plot showing the correlation between loci on each chromosome and FFA 20:4. Detailed information on the *p* values is given in Table S3 (Supporting Information), and 13 loci were found to be related to FFA 20:4 at the suggestive or significant genome‐wide thresholds. The Q–Q plots are shown in Figure S2 (Supporting Information), and Figure S2A (Supporting Information) illustrates that the analysis models of FFA 20:4 were reasonable. Chromosome 11 was the most relevant for FFA 20:4. According to the local Manhattan plot (Figure 2B), the association of the FFA 20:4 level with chromosome 11 was primarily driven by rs174559 (*p* = 2.30 × 10^−10^). Rs174559 is in the region of FADS1 (fatty acid desaturase 1) gene. The alleles at rs174559 are alleles G and A, with A being the major allele and G being the minor allele (minor allele frequency (MAF) = 0.39). The minor allele was related to an increased level of FFA 20:4 (β = 0.206).

The GWAS results of pipecolinic acid and leucine/isoleucine are also shown in Figure 2. Specific information on important SNPs related to pipecolinic acid and leucine/isoleucine at the suggestive or significant genome‐wide thresholds is summarized in Table S3 (Supporting Information). The rs535514 SNP on chromosome 1 is in the region of Cholinergic Receptor Muscarinic 3 (CHRM3) gene, which is related to insulin secretion.^[^ ^9^ ^]^ As shown in Figure 2D, allele C of rs535514 is related to an increased pipecolinic acid level (*p* = 5.40 × 10^−7^, β = 0.157, MAF = 0.42). Rs72487966 (SNP on chromosome 16) is located in the WW domain containing oxidoreductase (WWOX) gene region, which is a risk gene for T2D.^[^ ^10^ ^]^ As shown in Figure 2F, allele G of rs72487966 is related to an increased leucine/isoleucine level (*p* = 2.92 × 10^−6^, β = 0.411, MAF = 0.03). We also attempted to conduct two‐sample Mendelian randomization (MR) analysis to evaluate whether there are causal relationships between pipecolinic acid or leucine/isoleucine and T2D. Based on the meta‐analyses using summary statistics of the Dongfeng‐Tongji cohort, and the publicly available results of the KORA cohort and the Twins UK cohort,^[^ ^11^ ^]^ two‐sample MR analysis of leucine/isoleucine levels was conducted, and the OR per SD genetically predicted difference was scaled using an inverse‐variance‐weighted method. As a result, the relationship between leucine/isoleucine and T2D was proven to be causal (OR = 0.004, 95% CI 1.001–1.006, *p* = 0.001).

## 3 Discussion

In this nested Chinese prospective cohort, an untargeted ultra‐performance liquid chromatography‐mass spectrometry (UPLC‐MS)‐based metabolic profiling of serum samples was performed and integrated with genomics. To the best of our knowledge, this is one of the most comprehensive metabolomics studies conducted on Chinese participants without diabetes at baseline to reveal the significant metabolic changes before the onset of T2D and predict the risk of future T2D. According to our results, 206 metabolites were identified. Among them, 81 metabolites, including carnitines, amino acids, fatty acids, organic acids, and lipids were significantly changed before T2D onset. Eight metabolites related to future T2D risk were defined as a CMB for T2D prediction. Among them, four metabolites (LPC 18:2, 1,5‐anhydro‐D‐glucitol, FFA 20:4 and leucine/isoleucine) were reported in other cohort studies^[^ ^12^, ^13^, ^14^, ^15^ ^]^ and validated in our Chinese prospective cohort study. Four of them (pipecolinic acid, carnitine C14:0, epinephrine and PE 34:2) were first reported to be significantly associated with future T2D risk in a prospective cohort study. The AUCs of CMB were 0.708 and 0.687 in the discovery and validation sets, respectively, indicating that CMB had an acceptable predictive ability for the onset of T2D. The combination of CMB and CCB significantly improved the predictive ability of CCB from 0.755 to 0.773 in the discovery set and from 0.772 to 0.797 in the validation set.

In this study, before the onset of T2D, the levels of most LPEs and LPCs were decreased, while the levels of PCs and PEs were increased in the case group (Figure 1A). LPCs and LPEs are primarily derived from PCs and PEs by the action of phospholipase A.^[^ ^16^ ^]^ It was reported that the levels of phospholipids were significantly changed in diabetic patients.^[^ ^17^ ^]^ The significant changes of those phospholipids in our study hint that abnormal phospholipid metabolism appears before the onset of T2D. It is worth mentioning that as a member of CMB, a lower level of LPC 18:2 was found to be associated with future T2D risk in a European cohort,^[^ ^12^ ^]^ which was consistent with our results in the Chinese cohort. Furthermore, PE 34:0 was first reported to be associated with future T2D risk, and defined as a discriminant marker in our study.

FFAs are important energy substances that can be transferred into mitochondria for β‐oxidation with the help of carnitine. In our results, the levels of FFAs and acylcarnitines were significantly increased before T2D occurred. Most of them were significantly positively correlated with the development of T2D (values of ORs greater than 1 and values of multivariable‐adjusted *p* values less than 0.05). Among them, FFA 20:4 and carnitine C14:0 were also defined by binary logistic regression as predictors of T2D. According to published studies, the levels of FFAs and acylcarnitines are significantly increased in T2D patients.^[^ ^18^, ^19^ ^]^ FFA 20:4 was reported to be positively associated with future T2D in a French prospective cohort.^[^ ^13^ ^]^ Baseline carnitine C14:0 was also analyzed in another Chinese prospective cohort of T2D, but neither significant differences between the case group and the control group nor a significant relationship with the risk of future T2D was found.^[^ ^20^ ^]^ Furthermore, we calculated the ratio of carnitine C4:0/carnitine C3:0, which can indicate the activity of short‐chain acyl‐CoA dehydrogenase (SCAD).^[^ ^21^ ^]^ SCAD is the rate‐limiting enzyme of electron transport, which is the initial reaction in mitochondrial β‐oxidation.^[^ ^22^ ^]^ In our case group, the ratio of carnitine C4:0/carnitine C3:0 was significantly increased (*p* < 0.001), indicating the activity of SCAD was decreased. This demonstrated that before the occurrence of T2D, β‐oxidation of fatty acids may have been impaired. Notably, it was reported that incomplete fatty acid β‐oxidation can contribute to insulin resistance, which plays an important role in the pathogenesis of T2D.^[^ ^23^ ^]^

Furthermore, according to our mGWAS results, the most relevant SNP of FFA 20:4 was rs174559 (SNP on chromosome 11), which is in the region of FADS1 gene (Figure 2A,B). Some other SNPs mapping to the FADS1 gene were reported to be significantly associated with FFA 20:4.^[^ ^24^ ^]^ FADS1 encodes an important enzyme in the metabolism of unsaturated fatty acids.^[^ ^25^ ^]^ According to the pathway analysis in this study (Figure 1C), a significant disturbance in the biosynthesis of unsaturated fatty acids existed before the onset of T2D. FADS1 was also reported to be associated with an increased risk of T2D in a Japanese study.^[^ ^26^ ^]^

Pipecolinic acid was decreased in the case group in our study and significantly negatively associated with future T2D risk. Pipecolinic acid was also analyzed in a prospective cohort of T2D patients in a Mediterranean population, but no significant association between the baseline level of pipecolinic acid and T2D risk was found (*p* > 0.05).^[^ ^27^ ^]^ This is the first time that the baseline level of pipecolinic acid was found to be significantly related to future T2D risk. Furthermore, rs535514 was related to the pipecolinic acid level based on the mGWAS results, and rs535514 was in the regions of the CHRM3 gene (Figure 2D). The CHRM3 gene encodes the cholinergic receptor muscarinic 3, which can interfere with the control of the hypothalamus to regulate appetite. Notably, an association between CHRM3 and the risk of early occurrence of T2D was found in a Pima Indian population.^[^ ^28^ ^]^ CHRM3 is a gene related to the stimulation of insulin secretion.^[^ ^9^ ^]^ Together with the cholinergic receptor muscarinic 3, acetylcholine is capable of regulating insulin secretion,^[^ ^9^ ^]^ controlling islet cell proliferation and maintaining the glucose levels.^[^ ^29^ ^]^

The levels of amino acids in serum were significantly increased and positively related to future T2D, including branched‐chain amino acids (leucine/isoleucine), aromatic amino acids (phenylalanine and tyrosine), lysine and methionine. According to previous studies,^[^ ^30^, ^31^ ^]^ amino acid metabolism is closely related to T2D. For example, aromatic amino acids and branched‐chain amino acids were reported to be correlated with insulin resistance.^[^ ^30^ ^]^ Furthermore, high levels of branched amino acids were confirmed as risk factors for incident diabetes in a Japanese cohort.^[^ ^14^ ^]^ Branched amino acids were also chosen as predictors of future diabetes.^[^ ^31^ ^]^ In our work, leucine/isoleucine was significantly positively related to future T2D risk and selected as a marker for predicting the occurrence of diabetes, which is consistent with the previously mentioned article. According to the mGWAS results, a SNP (rs72487966) in the WWOX gene was related to leucine/isoleucine (Figure 2F). The WWOX gene is related to HOMA‐β,^[^ ^32^ ^]^ can regulate glucose metabolism^[^ ^33^ ^]^ and was found to be a susceptibility gene for diabetes.^[^ ^10^ ^]^ Moreover, according to the results of two‐sample MR analysis, the relationship between leucine/isoleucine and T2D was causal, and the elevated concentration of leucine/isoleucine increased the risk of T2D, which has been mutually confirmed with other published cohort studies.^[^ ^11^ ^]^ This indicates that these metabolic pathways that produce leucine/isoleucine may contribute to the development of T2D.

To the best of our knowledge, this study reports correlations between pipecolinic acid and CHRM3, and between leucine/isoleucine and WWOX for the first time. The new association might provide new insights into the pathogenesis of T2D. However, the correlations were suggestive, and more validation studies are needed.

Epinephrine and 1,5‐anhydro‐D‐glucitol were two of the metabolites that made up the CMB in our study. Epinephrine can increase endogenous glucose production.^[^ ^34^ ^]^ The lower 1,5‐anhydro‐D‐glucitol level has been clinically used as a marker of the increase in blood glucose levels.^[^ ^35^ ^]^ According to our results, a significant increase in epinephrine and a significant decrease in 1,5‐anhydro‐D‐glucitol preexisted before the occurrence of T2D (Figure 1). A prospective study in Shanghai, China, reported that 1,5‐anhydro‐D‐glucitol was negatively related to the risk of future T2D.^[^ ^15^ ^]^ However, to the best of our knowledge, this is the first report of a positive association between adrenaline and future T2D risk.

However, some limitations existed in our study. The participants were all Chinese individuals from Wuhan and were relatively old, so more research is needed to verify whether our research results are applicable to other age groups and other ethnic groups. In addition, the results of this study should be independently replicated in cohorts from different centers for verification.

In summary, we discovered metabolic changes that occurred prior to the onset of T2D and eight risk‐related metabolites that can be used to predict future T2D case in advance. Four metabolites among them were reported for the first time to be associated with future diabetes risk. The FADS1, CHRM3 and WWOX genes were found to be associated with T2D‐related metabolites. Furthermore, the relationship between increased levels of leucine/isoleucine and an increased risk of T2D was proven to be causal. Although further biofunctional validation studies are needed, our findings complement the current understanding of diabetes onset and can provide a basis for further mechanistic studies.

## 4 Experimental Section

##### Study Design and Participants

**Figure 3:**
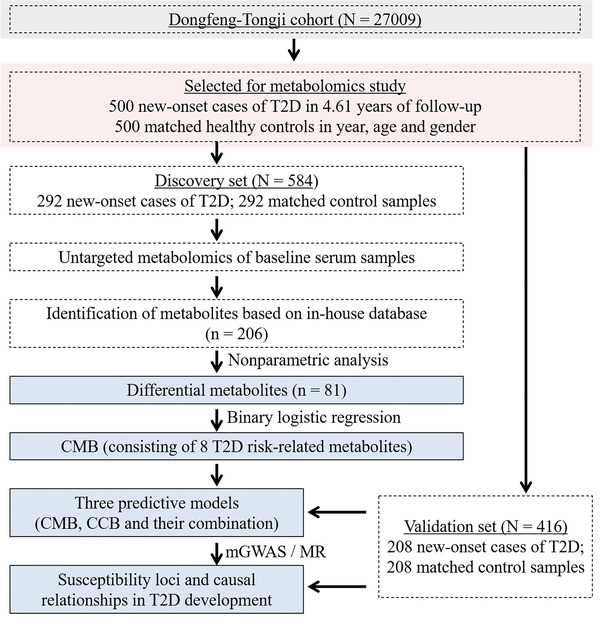
Flowchart of the study.

The flowchart of the study is shown in **Figure** **3**. Participants were from the Dongfeng‐Tongji cohort.^[^ ^7^ ^]^ All participants were retired employees of Dongfeng Motor Corporation with a mean age of 63 years. From September 2008 to June 2010, baseline serum samples were collected from 27009 individuals. All participants completed a lifestyle questionnaire and underwent a physical examination. In 2013, 25978 participants underwent a follow‐up examination, which accounted for 96.2% of all participants. Written informed consent was provided by all participants involved in this study, and approval of the research protocol (No. 2012(10)) was provided by the Ethics and Human Subjects Committee of Tongji Medical College, Huazhong University of Science & Technology.

After 4.61 ± 0.15 years of follow‐up, a total of 1039 eligible participants developed incidents in the Dongfeng‐Tongji cohort, among whom 500 new‐onset T2D subjects, excluding participants with cardiovascular disease or cancer, were selected as the case group. T2D was diagnosed if any of the following three criteria were met:^[^ ^36^ ^]^ 1) fasting blood glucose level higher than 7.0 mmol L^−1^; 2) hemoglobin A1c level higher than 6.5%; and 3) self‐reported diagnosis of T2D. Healthy subjects (*n* = 500) matched 1:1 by age and sex at baseline were selected as the control group. A total of 292 pairs of cases and matched control samples were randomly selected as the discovery set. The remaining 208 pairs of case and matched control samples were used as the validation set.

Untargeted UPLC‐MS‐based metabolic profiling of baseline fasting serum samples from the 1000 participants was performed. A Waters ACQUITY UPLC system (Waters Corp., Milford, MA, USA) was used for the separation of metabolites. For positive ion mode analysis, an ACQUITY UPLC BEH C8 (2.1 mm × 50 mm, 1.7 µm particle size, Waters Corp.) column and a TripleTOF 5600 mass spectrometer (AB SCIEX, Framingham, MA, USA) were used. For the negative ion mode analysis, an ACQUITY UPLC BEH T3 (2.1 mm × 50 mm, 1.8 µm particle size, Waters Corp.) column and a Q Exactive HF mass spectrometer (Thermo Fisher Scientific, Bremen, Germany) were used. The UPLC‐MS method was specifically designed for large‐scale metabolomics studies to assure the robustness and repeatability.^[^ ^37^ ^]^ Differential metabolites between the groups with or without the onset of diabetes were identified in the discovery set, and the metabolites used as the CMB were selected from them. The optimal clinical parameters were selected as the CCB and combined with the CMB to create as a combination predictive model. The predictive ability of each model for T2D onset was assessed by the AUC. In addition, the predictive abilities of the three models were further evaluated in the validation set. Genotyping of SNPs of the participants was performed. Furthermore, a metabolome‐genome‐wide association study was performed to find associations between T2D‐related metabolites and gene loci. Mendelian randomization (MR) analysis was conducted to scale possible causal relationships.

##### Statistical Analysis

Detailed information on the preparation of serum samples and the LC‐MS methods for metabolomics analysis are shown in the Supporting Information. According to the in‐house database,^[^ ^8^ ^]^ metabolites were identified by retention time, m/z value and MS/MS information. The peak areas of metabolites were adjusted by internal standards. The identified metabolites whose RSD below 30% in QCs were used for subsequent data analysis. Nonparametric tests were conducted by Multi‐Experiment Viewer software (version MEV 4.7.4)^[^ ^38^ ^]^ to identify significantly changed metabolites and clinical parameters (*p* < 0.05, FDR < 0.1) between the case and control groups. Pathway analysis of significantly changed metabolites was conducted by using MetaboAnalyst 4.0^[^ ^39^ ^]^ (Xia Lab, McGill University, Montreal, QC, Canada; https://www.metaboanalyst.ca/). All of the following analyses were conducted by IBM SPSS Statistics for Windows (version 25.0, IBM Corp, Armonk, New York, USA). Odds ratios and 95% confidence intervals were calculated by the Cox regression model. Binary logistic regressions were carried out to define clinical biomarkers and metabolic biomarkers. Receiver operating characteristic (ROC) curves were plotted to show the ability to differentiate between the controls and cases. Detailed information on the genotyping of SNPs of participants and genotype–metabolite association analysis is shown in the Supporting Information. As a result, ≈81 million SNP markers were obtained. Genotype‐metabolite association tests were performed by an SE‐weighted meta‐analysis with METAL (https://genome.sph.umich.edu/wiki/METAL_Documentation). The mendelian randomization‐based website (http://www.mrbase.org/) was used to conduct two‐sample MR analysis.

## Conflict of Interest

The authors declare no conflict of interest.

## Author Contributions

Y.O. and G.Q. contributed equally to this work. G.X. and T.W. conceived and designed the study, interpreted the data, critically revised the manuscript for important intellectual content and approved the version to be published. Y.O. and G.Q. contributed equally to this work. Y.O. and G.Q. performed data acquisition and statistical analyses. Y.O. wrote the manuscript. X.Z. interpreted the data, and critically revised the manuscript for important intellectual content, and approved the version to be published. B.Z., D.F., X.Q., W.L., and X.L. contributed to statistical analyses. W.J., D.Y., and Q.W. contributed to data acquisition.

## Supporting information

Supporting Information

## Data Availability

Research data are not shared.
