# Supplementary material for: Metabolome‐Genome‐Wide Association Study (mGWAS) Reveals Novel Metabolites Associated with Future Type 2 Diabetes Risk and Susceptibility Loci in a Case‐Control Study in a Chinese Prospective Cohort
Source: Glob Chall. 2021 Mar 23;5(4):2000088. doi: 10.1002/gch2.202000088 (PMC8025395; doi:10.1002/gch2.202000088)
Supplement: Supplementary file 1 — Supporting Information [file GCH2-5-2000088-s001.pdf]

© 2021 Wiley-VCH GmbH

Global Challenges
Open Access

Supporting Information

for *Global Challenges,* DOI: 10.1002/gch2.202000088

Metabolome-Genome-Wide Association Study (mGWAS) Reveals Novel Metabolites Associated with Future Type 2 Diabetes Risk and Susceptibility Loci in a Case-Control Study in a Chinese Prospective Cohort

Yang Ouyang, Gaokun Qiu, Xinjie zhao, Benzhe Su, Disheng Feng, Wangjie Lv, Qiuhui Xuan, Lichao Wang, Di Yu, Qingqing Wang, Xiaohui Lin, Tangchun Wu, and Guowang Xu**

Supporting Information

Metabolome-genome-wide association study (mGWAS) reveals novel metabolites associated with future type 2 diabetes risk and susceptibility loci in a case-control study in a Chinese prospective cohort

Yang Ouyang[1,2#], Gaokun Qiu[3#], Xinjie zhao[1], Benzhe Su[4], Disheng Feng[1,2], Wangjie Lv[1,2], Qiuhui Xuan[1,2], Lichao Wang[1,2], Di Yu[1,2], Qingqing Wang[1,2], Xiaohui Lin[4], Tangchun Wu[3*], Guowang Xu[1,2]*

[1] CAS Key Laboratory of Separation Science for Analytical Chemistry, Dalian Institute of Chemical Physics, Chinese Academy of Sciences, 457 Zhongshan Road, Dalian 116023, China.

[2] University of Chinese Academy of Sciences, Beijing 100049, China.

[3] MOE Key Lab of Environment and Health, School of Public Health, Tongji Medical College, Huazhong University of Science & Technology, Wuhan 430030, Hubei, China.

[4]School of Computer Science & Technology, Dalian University of Technology, Dalian 116024, China.

#: equal contribution.

*: Corresponding authors:

Prof. Dr. Guowang Xu, CAS Key Laboratory of Separation Science for Analytical Chemistry, Dalian Institute of Chemical Physics, Chinese Academy of Sciences, Dalian 116023, China. Tel. / Fax: 0086-411-84379530. E-mail: xugw@dicp.ac.cn.

Prof. Tangchun Wu, MOE Key Lab of Environment and Health, School of Public Health, Tongji Medical College, Huazhong University of Science & Technology, Wuhan 430030, Hubei, China. E-mail: wut@tjmu.edu.cn.

Metabolomics methods

Genotype–metabolite association analysis

Supplementary Tables:

Table S1. ORs per SD increment in clinical parameters in the discovery set and the validation set

Table S2. Detailed information on ORs of metabolic biomarkers in the discovery set

Table S3. Associations of single nucleotide polymorphisms with metabolites

Supplementary Figures:

Figure S1. Relative standard deviation (RSD) distribution of identified metabolites in quality control samples in the discovery set (A) and the validation set (B).

Figure S2. Q-Q plots of FFA 20:4 (A), pipecolinic acid (B) and leucine/isoleucine (C).

Metabolomics method

Materials and reagents

Acetonitrile and methanol for liquid chromatography were obtained from Merck (Darmstadt, Germany). Ammonium bicarbonate and formic acid were purchased from Sigma-Aldrich (St. Louis, MO, USA). Ultrapure water was produced by a Milli-Q water purification system (Millipore, Billerica, MA, USA). Impact Protein Precipitation 96-well (2 mL Square Well) plates (Phenomenex, Torrance, CA, USA) were used for the pretreatment of serum samples. Internal standards (ISs) were purchased form Avanti Polar Lipids (Alabaster, AL, USA). The concentrations of ISs were as follows: tryptophan-d5 (4.250 μg mL^{-1}); phenylalanine-d4 (3.612 μg mL^{-1}); cholic acid-d4 (1.854 μg mL^{-1}); chenodeoxycholic acid-d4 (1.485 μg mL^{-1}); palmitic acid-d3 (2.500 μg mL^{-1}); stearic acid-d3 (2.500 μg mL^{-1}); decanoylcarnitine-d3 (0.100 μg mL^{-1}); palmitoylcarnitine-d3 (0.150 μg mL^{-1}); lysophosphatidylcholine 19:0 (0.750 μg mL^{-1}); phosphatidylcholine 38:0 (0.976 μg mL^{-1}); sphingomyelin 12:0 (0.500 μg mL^{-1}).

Serum sample preparation

Fifty microliters of serum were taken from each sample for analysis. The serum was added to a 96-well plate, and a 4-fold volume of methanol containing the internal standards was added. After vortexed for 10 min at room temperature, the mixture was centrifuged (4 °C, 10 min, 500 g) to remove the protein. The filtrate was lyophilized and stored at -80 °C. Prior to LC-MS analysis, each sample was resuspended in 50 µL of water/methanol (80:20, v/v), vortexed for 10 min, and centrifuged for 15 min (10 °C, 800 g). The samples were randomly sorted and the total sample size was 1000. The quality control (QC) samples were obtained by mixing the laboratory serum

samples and were processed according to the above methods. One QC sample was analyzed for every 10 real serum samples to assess the stability of the analysis.

LC-MS methods

A Waters ACQUITY UPLC system (Waters Corp, Milford, MA, USA) coupled to a TripleTOF 5600 mass spectrometer (AB SCIEX, Framingham, MA, USA) operated in positive ion mode was used for metabolomics analysis. The chromatography column used was an ACQUITY UPLC BEH C8 (2.1 mm × 50 mm, 1.7 μm particle size, Waters Corp., Milford, MA USA) column. Phase A was water with 0.1% formic acid. Phase B was acetonitrile with 0.1% formic acid. A short gradient (12 min) was used to reduce the analysis time. Specific LC-MS conditions were described in our previous work [1]. The starting composition of the mobile phase was 5% B and was maintained for 0.5 min. Then, it was linearly increased to 40% at 2.0 min and then to 100% at 8.0 min. After 2.0 min, it was returned to 5% B in 0.1 min and maintained to 12 min for re-equilibration. The flow rate was set to 0.4 mL/min, and the injection volume was 5 μL. The column temperature was 60 °C. The ion source temperature was set to 550 °C. The ion spray voltage was 5.5 kV. The scan range was m/z 80-1000. The information-dependent acquisition mode was used to obtain the MS^2 spectra, and the scan range was m/z 60-1000. The collision energy was set at 40 V, and the collision energy spread was 20 V.

For negative ion mode analysis, the MS system used was a Q Exactive HF mass spectrometer (Thermo Fisher Scientific, Bremen, Germany). The chromatography column used was an ACQUITY UPLC BEH T3 (2.1 mm × 50 mm, 1.8 μm particle size, Waters Corp., Milford, MA, USA) column. The mobile phases C and D were water and H_2O/CH_3OH (5:95, v/v) (both containing 6.5 mmol/L NH_4HCO_3),

respectively. The gradient was similar to that used in the positive ion mode, except that the starting composition was 2% D and the re-equilibration composition was 2% D. The other LC parameters were the same as those used in the positive ion mode. For MS, the capillary temperature was 300 °C. The flow rates of the auxiliary gas and sheath gas were 10 and 45 (in arbitrary units), respectively. The auxiliary gas heater temperature was set to 350 °C. The S-lens RF level was set to 50. The ion spray voltage was set to 3.0 kV. Full scan MS/data-dependent MS/MS (ddMS2) mode was used. The resolutions of full scan MS and ddMS2 were 120,000 and 30,000, respectively. The scan range was m/z 70–1050.

Raw data of metabolomics obtained in positive ion mode were processed by SIEVE software (version 2.2, Thermo Fisher Scientific, San Jose, CA, USA). Raw data obtained in negative ion mode were processed by MarkerView software (version 1.2.1, AB SCIEX, Concord, ON, Canada).

Genotype–metabolite association analysis

Genotyping of SNPs of participants was performed on an Affymetrix Genome-Wide Human SNP Array 6.0 Chips or Illumima Infinium OmniZhongHua-8 Chips. We removed any SNPs with a missing call rate > 5%, MAF < 1%, *p*(HWE) < 10^{-5} or flipping strands, resulting in 703,302 SNPs in the Illumina dataset and 549,196 SNPs in the Affymetrix dataset. Using the 1000 Genomes Project ALL Phase 3 Integrated Release Version 5 Haplotypes (05/02/2013) as the reference panel, we imputed untyped SNPs according to linkage disequilibrium information for study samples of the Illumina and Affymetrix datasets, achieving a total of approximately 81 million SNP markers. To obtain a larger sample size, another 1000 participants in an extended cardiac disease study (500 cases in five years of follow-up, 500 matched

controls) from the same Dongfeng-Tongji cohort were selected to perform the same genetic sequencing and metabolomics analysis. Genotype-metabolite association tests were performed within each of the two datasets (2 × 1000 persons) separately using logistic regression under an additive model with SNPTEST (https://mathgen.stats.ox.ac.uk/genetics_software/snptest/snptest.html) by adjusting age, sex, and the top five principal components of the genotypes to account for population stratification, followed by an SE-weighted meta-analysis with METAL (https://genome.sph.umich.edu/wiki/METAL_Documentation). The genome-wide significant *p*-value threshold was 5×10^{-8}, while the suggestive threshold was 5×10^{-6}. The Mendelian randomization (MR)-based website (http://www.mrbase.org/) [2] was used to conduct two-sample MR analysis based on the meta-analyses using summary statistics of the Dongfeng-Tongji cohort, and the publicly available results of the KORA cohort and the TwinsUK cohort[3-5]. The odds ratio (OR) was scaled by using an inverse-variance-weighted method[6] to represent a genetically predicted difference per SD in metabolite levels.

References

[1] Y. Ouyang, H. Tong, P. Luo, H. Kong, Z. Xu, P. Yin, G. Xu, Talanta **2018**, *185*, 483.

[2] MR-base website. http://www.mrbase.org/.

[3] L. A. Lotta, R. A. Scott, S. J. Sharp, S. Burgess, J. a. Luan, T. Tillin, A. F. Schmidt, F. Imamura, I. D. Stewart, J. R. B. Perry, L. Marney, A. Koulman, E. D. Karoly, N. G. Forouhi, R. J. O. Sjögren, E. Näslund, J. R. Zierath, A. Krook, D. B. Savage, J. L. Griffin, N. Chaturvedi, A. D. Hingorani, K. T. Khaw, I. Barroso, M. I. McCarthy, S. O'Rahilly, N. J. Wareham, C. Langenberg, PLOS Medicine **2016**, *13*,

e1002179.

[4] Y. Hu, H. Li, L. Lu, A. Manichaikul, J. Zhu, Y.-D. I. Chen, L. Sun, S. Liang, D. S. Siscovick, L. M. Steffen, M. Y. Tsai, S. S. Rich, R. N. Lemaitre, X. Lin, Human Molecular Genetics **2016**, *25*, 1215.

[5] W. Guan, B. T. Steffen, R. N. Lemaitre, J. H. Y. Wu, T. Tanaka, A. Manichaikul, M. Foy, S. S. Rich, L. Wang, J. A. Nettleton, W. Tang, X. Gu, S. Bandinelli, I. B. King, B. McKnight, B. M. Psaty, D. Siscovick, L. Djousse, Y.-D. I. Chen, L. Ferrucci, M. Fornage, D. Mozafarrian, M. Y. Tsai, L. M. Steffen, Circulation: Cardiovascular Genetics **2014**, *7*, 321.

[6] S. Burgess, F. Dudbridge, S. G. Thompson, Statistics in Medicine **2016**, *35*, 1880.

Table S1. ORs per SD increment in clinical parameters in the discovery set and the validation set

Variables	Discovery set		Validation set	
	OR (95% CI)	*p*	OR (95% CI)	*p*
Age (years)	0.896 (0.679-1.182)	0.438	0.847 (0.602-1.192)	0.341
Men sex, No. (%)	/	/	/	/
BMI (kg m^{-2})	1.181 (1.115-1.250)	< 0.001	1.221 (1.136-1.313)	< 0.001
Smoking status, No. (%)				
Current smoker	1.051 (0.549-2.012)	0.228	1.187 (0.474-2.970)	0.279
Former smoker	0.750 (0.454-1.239)		0.715 (0.392-1.303)	
Never smoker	1.000 Ref		1.000 Ref	
Drinking status, No. (%)				
Current drinker	1.974 (0.752-5.179)	0.885	0.793 (0.335-1.281)	0.293
Former drinker	1.056 (0.679-1.641)		0.764 (0.456-1.281)	
Never drinker	1.000 Ref		1.000 Ref	
Physical activity, No. (%)	1.448 (0.849-2.468)	0.174	1.138 (0.624-2.075)	0.674
Systolic blood pressure (mmHg)	1.012 (1.002-1.021)	0.016	1.007 (0.997-1.018)	0.187
Diastolic blood pressure (mmHg)	1.021 (1.006-1.036)	0.007	1.004 (0.986-1.023)	0.635
HDL cholesterol (mmol L^{-1})	0.623 (0.425-0.913)	0.015	0.625 (0.386-1.011)	0.055
LDL cholesterol (mmol L^{-1})	1.070 (0.864-1.325)	0.536	0.964 (0.745-1.247)	0.778
Triglycerides (mmol L^{-1})	1.743 (1.380-2.201)	< 0.001	1.454 (1.143-1.849)	0.002
Fasting glucose (mmol L^{-1})	4.178 (2.876 -6.069)	< 0.001	4.921 (3.116-7.771)	< 0.001

[a)] ORs per SD increment and *p* values were calculated by Cox regression, and clinical indictors were adjusted for age and sex (except age and gender).

Table S2. Detailed information on ORs of metabolic biomarkers in the discovery set

Metabolites	Trend	OR[1*]	p^{1*}	OR[2*]	p^{2*}	Q1[Δ]	Q2[Δ]	Q3[Δ]	Q4[Δ]	p^{Δ}
Pipecolinic acid	Down	0.736 (0.609-0.89)	0.002	0.731 (0.597-0.896)	0.002	1.000 Ref	0.581 (0.348-0.970)	0.507 (0.299-0.859)	0.451 (0.268-0.760)	0.002
1,5-Anhydro-D-Glucitol	Down	0.769(0.646-0.916)	0.003	0.808 (0.670-0.973)	0.025	1.000 Ref	0.799 (0.470-1.360)	0.551 (0.333-0.911)	0.601 (0.358-1.008)	0.024
LPC 18:2	Down	0.690(0.569-0.837)	< 0.001	0.846 (0.687-1.042)	0.116	1.000 Ref	0.947 (0.575-1.559)	0.663 (0.391-1.124)	0.657 (0.379-1.140)	0.085
Carnitine 14:0	Up	1.401(1.170-1.677)	< 0.001	1.392 (1.141-1.698)	0.001	1.000 Ref	1.191 (0.7231.962)	2.126 (1.208-3.739)	2.540 (1.466-4.404)	< 0.001
PE 34:2	Up	1.423(1.189-1.705)	< 0.001	1.341 (1.103-1.631)	0.003	1.000 Ref	1.011 (0.596-1.715)	1.293 (0.763-2.191)	2.235 (1.265-4.061)	0.004
FFA 20:4	Up	1.260(1.037-1.530)	0.020	1.229 (1.009-1.497)	0.041	1.000 Ref	1.482 (0.879-2.496)	1.149 (0.686-1.925)	3.206 (1.806-5.689)	< 0.001
Isoleucine/Leucine	Up	1.626(1.340-1.973)	< 0.001	1.427 (1.157-1.761)	< 0.001	1.000 Ref	1.580 (0.915-2.728)	2.201 (1.246-3.888)	2.907 (1.609-5.254)	< 0.001
Epinephrine	up	1.277 (1.073-1.520)	0.006	1.315 (1.084 – 1.595)	0.005	1.000 Ref	1.583 (0.947-2.647)	1.085 (0.644 -1.827)	2.312 (1.320-4.049)	0.020

[a)] ORs of metabolite biomarkers were obtained with conditional logistic models; [b)] * Metabolites defined as continuous variables were classified into two kinds: 1 was unadjusted and 2 was adjusted for age, sex, BMI, smoking status, drinking status and physical activity; [c)] Δ Metabolites defined as categorical variables and were adjusted for age, sex, BMI, smoking status, drinking status and physical activity.

Table S3. Associations of single nucleotide polymorphisms with metabolites*

Related metabolites	SNP	Nearest gene	Chr.	Position	Major/minor allele	MAF	β ± SE	*p*
	rs76670355	ZNF644	1	91480526	C/T	0.02	0.502 ± 0. 104	1.23×10^{-6}
	rs2501873	NR1I3	1	161204538	T/C	0.44	-0.145 ± 0. 031	2.30×10^{-6}
	rs76221478	RALGPS2	1	178882970	T/G	0.01	0.831 ± 0. 179	3.45×10^{-6}
	rs2202318	CNTN6	3	1108443	G/A	0.13	-0.221 ± 0. 048	$3.74 \times 10-^{6}$
	rs181856708	CDH12	5	21799595	T/A	0.01	-1.147 ± 0. 219	1.70×10^{-7}
	rs148631912	PTPRD	9	9982478	G/GTGTGTGTGT	0.23	0.213 ± 0. 044	9.95×10^{-7}
FFA 20:4	rs7918978	LOC101927824	10	3095308	T/C	0.26	-0.159 ± 0. 035	4.43×10^{-6}
	rs17306253	LOC105378404	10	87302288	C/G	0.04	0.370 ± 0. 078	2.12×10^{-6}
	rs199969886	DAGLA	11	61456980	CT/C	0.31	-0.201 ± 0. 039	3.05×10^{-7}
	rs174559	FADS1	11	61581656	A/G	0.39	0.206 ± 0. 033	2.30×10^{-10}
	rs11219419	OR10G7	11	123909073	G/C	0.32	-0.155 ± 0. 034	4.75×10^{-6}
	rs151282290	CRACR2A	12	3732860	C/T	0.01	-1.789 ± 0. 385	3.41×10^{-6}
	rs4818944	TSPEAR	21	46041718	A/G	0.24	0.174 ± 0. 037	1.97×10^{-6}
	rs181065252	S100PBP	1	33288928	C/T	0.01	0.934 ± 0. 182	2.93×10^{-7}
	rs111403249	CERS2	1	150949121	A/G	0.01	-2.943 ± 0. 596	7.91×10^{-7}
	rs535514	CHRM3	1	239971695	T/C	0.42	0.157 ± 0. 031	5.4×10^{-7}
	rs11722634	KCNIP4	4	21866760	T/C	0.01	-2.798 ± 0. 597	2.76×10^{-6}
	rs144093082	LINC02112	5	9887687	T/G	0.01	-2.697 ± 0. 573	2.47×10^{-6}
	rs74293705	SLCO4C1	5	101608386	A/G	0.02	-0.638 ± 0. 124	2.82×10^{-7}
	rs1407079	SOBP	6	107822332	G/A	0.12	-0.223 ± 0. 047	2.44×10^{-6}
pipecolinic acid	rs73166967	ATP6V0A4	7	138452440	C/T	0.03	0.534 ± 0. 114	2.65×10^{-6}
	rs13284095	TXNDC8	9	113089748	A/G	0.02	0.679 ± 0. 147	3.69×10^{-6}
	rs142133650	OVOS2	12	31349309	G/A	0.11	0.246 ± 0. 052	2.00×10^{-6}
	rs9524552	TGDS	13	95249907	C/T	0.01	2.250 ± 0. 464	1.22×10^{-6}
	rs76037355	GRAMD2A	15	72483401	T/C	0.01	-3.224 ± 0. 677	1.92×10^{-6}
	rs11085204	VAV1	19	6808762	C/T	0.12	-0.228 ± 0. 048	1.82×10^{-6}
	rs4801998	MIR521-2	19	54219769	A/G	0.38	-0.193 ± 0. 033	6.64×10^{-9}
	rs3746800	SLC52A3	20	742217	G/C	0.15	0.232 ± 0. 051	4.79×10^{-6}
	rs220308	UMODL1	21	43516589	G/T	0.50	0.141 ± 0. 031	4.58×10^{-6}
	rs11425021	LOC101928202	1	157273008	CA/C	0.17	0.238 ±0.045	1.56×10^{-7}
	rs76722445	TCEANC2	1	54544433	A/G	0.01	0.828 ± 0.174	2.03×10^{-6}
leucine/Isoleucine	rs10917520	TMCO4	1	20027049	A/C	0.29	-0.157 ± 0.032	6.23×10^{-7}
	rs368027712	ACTR3	2	114655164	T/C	0.01	0.744 ± 0.157	1.99×10^{-6}
	rs146001009	DPP10	2	115283960	A/G	0.01	-0.799 ± 0.169	2.28×10^{-6}
	rs200814804	LOC102724340	2	185094246	T/TA	0.01	1.034 ± 0.185	2.35×10^{-8}

rs147546513	ERC2	3	55675447	A/G	0.02	-0.510 ± 0.107	2.02×10^{-6}
rs141583750	KALRN	3	124000986	A/C	0.01	-1.017 ± 0.197	2.39×10^{-7}
rs189881113	PIK3CA	3	178906696	A/T	0.01	-0.923 ± 0.191	1.36×10^{-6}
rs187095574	TECRL	4	65142900	A/G	0.01	1.076 ± 0.213	4.40×10^{-7}
rs10080391	CDYL	6	4912441	T/C	0.11	0.244 ± 0.046	1.18×10^{-7}
rs186760583	SRRM3	7	75905518	A/C	0.02	0.501 ± 0.108	3.89×10^{-6}
rs79258342	USP47	11	11957205	A/G	0.02	-2.440 ± 0.490	6.24×10^{-7}
rs139827572	COL2A1	12	48392293	T/C	0.01	0.926 ± 0.200	3.57×10^{-6}
rs188931499	GDE1	16	19522578	C/G	0.01	0.850 ± 0.178	1.80×10^{-6}
rs72487966	WWOX	16	78432786	A/G	0.03	0.411 ± 0.088	2.92×10^{-6}
rs2288901	ILF3	19	10792573	T/C	0.02	-0.567 ± 0.120	2.08×10^{-6}
rs149426715	TEX33	22	37401436	T/C	0.02	0.711 ± 0.150	2.18×10^{-6}

[a)] *: SNP, single nucleotide polymorphisms; Chr., chromosome; MAF, minor allele frequency; β, effect size.

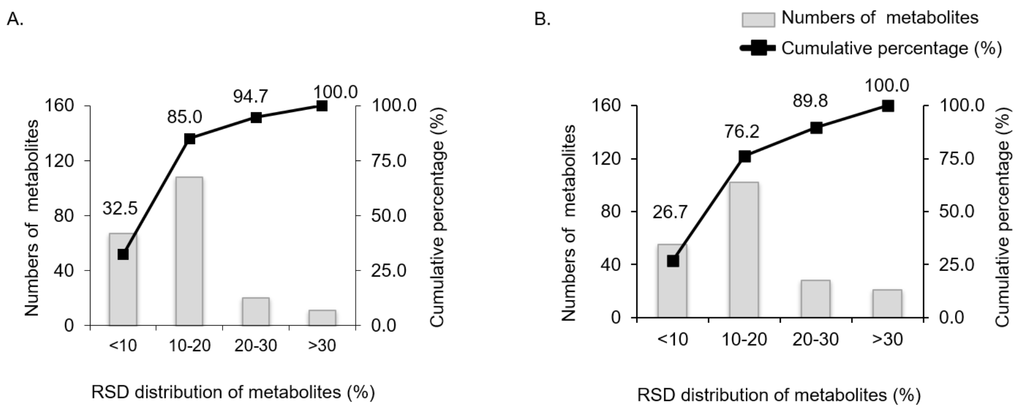

Figure S1. Relative standard deviation (RSD) distribution of identified metabolites in quality control samples in the discovery set (A) and the validation set (B).

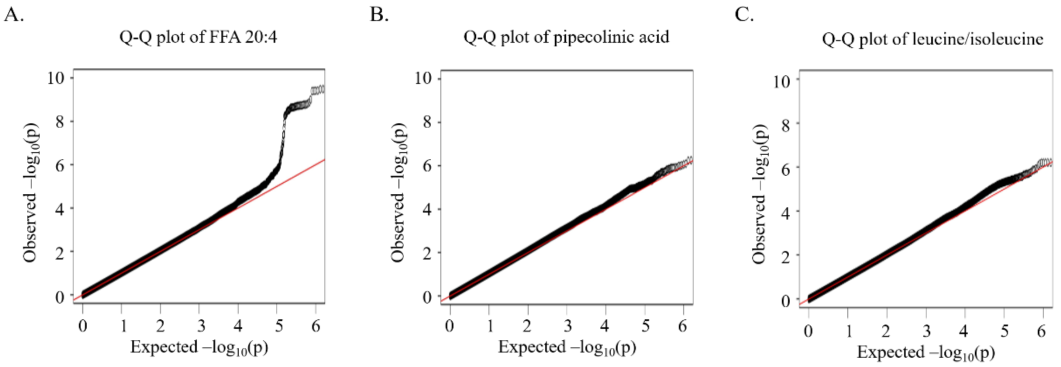

Figure S2. Q-Q plots of FFA 20:4 (A), pipecolinic acid (B) and leucine/isoleucine (C).
